# Induced pluripotent stem cells: Generation methods and a new perspective in COVID-19 research

**DOI:** 10.3389/fcell.2022.1050856

**Published:** 2023-01-17

**Authors:** Zahra Karami, Sharif Moradi, Akram Eidi, Masoud Soleimani, Arefeh Jafarian

**Affiliations:** ^1^ Department of Biology, Science and Research Branch, Islamic Azad University, Tehran, Iran; ^2^ Department of Stem Cells and Developmental Biology, Cell Science Research Center, Royan Institute for Stem Cell Biology and Technology, ACECR, Tehran, Iran; ^3^ Hematology and Cell Therapy Department, Faculty of Medical Sciences, Tarbiat Modares University, Tehran, Iran; ^4^ Department of Tissue Engineering and Applied Cell Science, School of Advanced Technologies in Medicine, Shahid Beheshti University of Medical Sciences, Tehran, Iran; ^5^ Iranian Tissue Bank and Research Center, Tehran University of Medical Sciences, Tehran, Iran

**Keywords:** transcription factors, pluripotency, reprogramming, drug screening, disease modeling, mRNA

## Abstract

Induced pluripotent stem cells (iPSCs) exhibit an unlimited ability to self-renew and produce various differentiated cell types, thereby creating high hopes for both scientists and patients as a great tool for basic research as well as for regenerative medicine purposes. The availability and safety of iPSCs for therapeutic purposes require safe and highly efficient methods for production of these cells. Different methods have been used to produce iPSCs, each of which has advantages and disadvantages. Studying these methods would be very helpful in developing an easy, safe, and efficient method for the generation of iPSCs. Since iPSCs can be generated from somatic cells, they can be considered as valuable cellular resources available for important research needs and various therapeutic purposes. Coronavirus disease 2019 (COVID-19) is a disease that has endangered numerous human lives worldwide and currently has no definitive cure. Therefore, researchers have been rigorously studying and examining all aspects of COVID-19 and potential treatment modalities and various drugs in order to enable the treatment, control, and prevention of COVID-19. iPSCs have become one of the most attractive and promising tools in this field by providing the ability to study COVID-19 and the effectiveness of drugs on this disease outside the human body. In this study, we discuss the different methods of generation of iPSCs as well as their respective advantages and disadvantages. We also present recent applications of iPSCs in the study and treatment of COVID-19.

## 1 Introduction

In recent years, major advances have been made in the field of stem cells and regenerative medicine (Liu et al., 2020). Cell-based therapy is considered one of the most promising methods in modern medicine (Aly, 2020). Accessibility of pluripotent stem cells (PSCs) with their vast potential for proliferation and differentiation provides new chances for basic research, disease modeling, drug discovery, and advancement of cell therapies (Martin, 2017). PSCs have two characteristics of self-renewal and pluripotency (Romito and Cobellis, 2016). Although only embryonic stem cells (ESCs) are genuinely pluripotent, differentiated cells can also be converted to a pluripotent state (Zakrzewski et al., 2019). Induced pluripotent stem cells (iPSCs) are PSCs produced by reprogramming somatic cells through overexpression of certain pluripotency markers (Ye et al., 2013; Moradi et al., 2019). iPSCs like ESCs can be preserved and expanded indefinitely *in vitro*, and they are able to differentiate into the derivatives of all three germ layers as well as germ cells that produce gametes (Romito and Cobellis, 2016; Moradi et al., 2019). As applying ESCs is accompanied with ethical and immunological problems, iPSCs have appeared as a hopeful solution. Because they are obtained from somatic tissues and cell sources for generating iPSCs are abundant, there are no or less ethical restrictions associated with iPSCs as opposed to ESCs (Moradi et al., 2019). Moreover, because iPSCs can be generated from the patients themselves, immune rejection is prevented (Liu et al., 2020). Kazutoshi Takahashi and Shinya Yamanaka generated the first lines of iPSCs in 2006 (Zahumenska et al., 2020). They reprogrammed mouse fibroblasts using four transcription factors, that is, OCT4, SOX2, KLF4, and c-MYC, into ESC-like cells which they designated iPSCs (Han et al., 2021a). These cells are similar to ESCs in characteristics such as morphology, pluripotency, and marker expression. While ESCs are obtained from the blastocyst᾽s inner cell mass (ICM), iPSCs can be derived from a wide range of different cellular sources using different reprogramming methods (Wang, 2021). In the earliest studies on iPSCs, the Yamanaka laboratory used different types of retroviruses to produce iPSCs, each carrying only one type of the transcription factor. This can lead to uncontrollable incorporation events accompanied by an enhanced probability of transgene reactivation, ineffective transgene silencing, and decreased reprogramming efficiency (Omole and Fakoya, 2018). To solve this problem, several non-integrative reprogramming techniques have been successfully developed to induce pluripotency in different somatic cell types (Steinle et al., 2019).

iPSC technology has extensive applications and great promise in production of patient-specific iPSC lines for 1) differentiation into the desired therapeutically relevant cell types for transplantation, modeling the pathophysiology of diseases to develop new therapies, drug screening for evaluating effectiveness and potential toxicity of drugs, developing personalized therapies, and the study of various genetic and epigenetic disorders (Shi et al., 2017; Bento et al., 2020).

The coronavirus disease 2019 (COVID-19), which is caused by the severe acute respiratory syndrome of corona-2 virus (SARS-CoV-2), has now become a pandemic, a major global threat that continues to spread. SARS-CoV-2 is an enveloped, positive-strand RNA virus. The mutation rate of RNA viruses, which causes virus evolution and genomic diversity, the ability of viruses to escape from the host immune system, and their resistance to antiviral drugs is very high. For these reasons, COVID-19 remains a major global challenge. The study of viral biology, virus–host interactions, and drug screening is a necessity for the rapid development and expansion of new prevention and treatment methods (Nolasco et al., 2020; Pachetti et al., 2020; Ahmadi and Moradi, 2021; Kanimozhi et al., 2021). Cellular and animal models are key platforms to study the pathogenesis of COVID-19 and the efficacy of drugs to treat SARS-CoV-2 infection. Cellular models have so far been used to evaluate the viral titer amounts of infected samples isolated from patients and the three-dimensional crystal structure of proteins by overexpressing specific SARS-CoV-2 proteins (Kanimozhi et al., 2021). The advent of cellular reprogramming technology and the production of iPSCs from somatic cells have made it possible to model and study human diseases, including COVID-19 (Esmail and Danter, 2021). Modeling COVID-19 and SARS-CoV-2 infection is of critical importance to interrogate the underlying molecular mechanisms mediating viral replication and pathogenesis and enabled the development of targeted therapeutic strategies against the virus (Estrada, 2020). Since human-induced pluripotent stem cells (hiPSCs) have the ability to produce a large variety of disease-relevant differentiated cell types, they are considered as a powerful research tool for disease modeling and drug screening (Nolasco et al., 2020). The global market of iPSCs is projected to increase from $2.8 billion in 2021 to $4.4 billion by 2026 with a compound annual growth rate (CAGR) of 9.3% for the period 2021–2026 (Fan, 2017). The present study describes different methods of iPSC generation and the pros and cons of each method. We also critically address various aspects of the process of reprogramming somatic cells to iPSCs and discuss the importance and applications of iPSCs in the study and treatment of the latest global health challenge, COVID-19.

## 2 How reprogramming takes place

The beginning of somatic cell reprogramming takes place through alterations of transcriptome and chromatin landscapes of a differentiated cell state to a partially reprogrammed, pluripotent state. Regulation of the binding of reprogramming transcription factors to pluripotency-related genomic sequences in somatic cells is frequently accomplished through changes in the chromatin structure under the influence of DNA methylation, histone modifications, and ATP-dependent chromatin remodeling events. The reprogramming factors typically form protein complexes with each other and create an interconnected self-sustained regulatory circuitry that activates and/or silences a large number of genes in the reprogramming cells and interact with other pluripotency factors to gradually establish a bonafide pluripotency state in the cells. In this circuitry, Oct4 and then Sox2 play the most pivotal functions (Al Abbar et al., 2020).

Reprogramming occurs in two stages. Initially, Oct4, Sox2, Klf4, and c-Myc (OSKM) bind to certain regions of chromatin that are not available to other (somatic cell) factors, leading to the rearrangement of the chromatin regions and turning the expression of numerous genes on or off. OSKM occupy many genomic sites, including sites that do not serve as binding sites for these factors in embryonic cells. In particular, c-MYC binds to the genomic regions with methylated H3K4, which is known to be an open-chromatin mark. By binding to enhancers and promoters determinant of somatic cell identity, OSKM promote the silencing of somatic genes. At the same time, OSKM bind to enhancers and promoters of pluripotency genes to induce their expression (Takahashi and Yamanaka, 2016). These collective regulatory processes drive the gradual reprogramming of the somatic cell gene expression profile to a gene expression signature typical of pluripotent cells.

## 3 Delivery of reprogramming factors into somatic cells

Various methods that have been designed to deliver reprogramming factors into somatic cells influence the reprogramming efficiency and quality of iPSCs (Stadtfeld and Hochedlinger, 2010). A plasmid or vector carrying transgenes is an essential part of gene delivery into cells. Plasmid DNA consists of an arbitrary transgene under the control of a promoter selected based on the required level of expression. The plasmid also contains a drug-resistant gene that can be used to monitor the cells that receive the plasmid and the source of bacterial multiplication. An effective method of gene delivery to target cells is a crucial factor for successful modification of gene expression. Chemical reagents such as Fugene HD, Gene Jammer, or Lipofectamine 2000 can be added directly to the culture medium to enable transfection of the plasmids into the cells, although this delivery strategy suffers from poor efficiency. As an alternative, customized electroporation devices such as Amaxa Nucleofector and Neon electroporation systems appear to increase the efficiency of the vector delivery. Finally, viral transduction strategies are used to overcome the inefficiency of chemical and/or electroporation methods (Fontes and Lakshmipathy, 2013).

## 4 Delivery methods

### 4.1 Integrative techniques

In comparison to non-integrative approaches, integrative techniques for gene delivery have superior delivery efficiency; however, they are less safe owing to the risk of insertional mutagenesis (Omole and Fakoya, 2018).

#### 4.1.1 Viral integrative vectors

##### 4.1.1.1 Retroviruses

In the first research on iPSCs, retroviral vectors were used to ectopically express reprogramming factors that were constantly integrated into the host cell genome. In this method, while at the end of the reprogramming process, retroviral transgenes are usually silenced, DNA and histone methyltransferases are activated, and therefore, the reprogramming process is often incomplete. Partially reprogrammed iPSC lines still require external factors and cannot activate the relevant endogenous genes. Moreover, in iPSC-derived somatic cells, the activity of viral transgene remainders or their reactivation can disrupt the normal developmental potential of the cells and cause tumors (Stadtfeld and Hochedlinger, 2010). Using retroviruses for the overexpression of transcription factors is a highly efficient and relatively facile method. Retroviruses only infect dividing somatic cells which allows them to successfully integrate their genomes into the host cell genome. Complete reprogramming occurs only when the endogenous genes associated with pluripotency are upregulated and the integrated transgenes are downregulated. Although retroviral vectors are usually silenced in ESCs and iPSCs, the inactivation of retroviral vectors is leaky and there is a possibility of transgene reactivation. Random integration of retroviruses into the host genome enhances the danger of insertional mutations (Omole and Fakoya, 2018), which highlights the fact that the most efficient methods for the delivery of reprogramming factors suffer from lower safety.

##### 4.1.1.2 Lentiviruses

Lentiviruses, which are single-stranded RNA viruses, are members of the Retroviridae family. They can effectively infect both dividing and non-dividing cells, making them powerful gene delivery tools. The lentiviral genome integrates into the host cell genome after reverse transcription. Because the integration of lentiviruses into the host genome yields long-term expression *in vitro*, they are mainly used when stable expression is required in target cells. This feature is not available with episomal vectors (Rapti et al., 2015). Transduction efficiency with lentiviruses is high, but there is a risk of insertional mutagenesis and multiple proviral integration, which can lead to abnormal alternative splicing and misplaced transcripts (Fontes and Lakshmipathy, 2013). The expression of lentiviral vectors can be controlled with the drug doxycycline, which reduces the risk of continuous transgene expression and allows the selection of completely reprogrammed iPSCs, because the reprogramming cells depend on the expression of exogenous factors and as soon as doxycycline is removed, cells stop proliferating. Lentiviral vectors are more efficient in infecting somatic cell types than retroviral vectors, and they can also be applied to express polycistronic cassettes encoding all four reprogramming factors, which enhance reprogramming efficiency (Stadtfeld and Hochedlinger, 2010).

### 4.2 Non-integrative viral vectors

The first integration-free iPSCs were generated by Stadtfeld et al. from mature mouse hepatocytes by the application of non-integrative adenoviruses in 2008 (Sridharan et al., 2009). Zhou and Freed generated iPSCs without transgene integration from human fibroblasts by applying adenoviral vectors in 2009. The production of iPSCs using adenoviral vectors has disadvantages such as the need for multiple viral infections, the laborious production of adenoviruses, and the low efficiency of the reprogramming procedure compared to the use of lentiviruses and retroviruses (Omole and Fakoya, 2018).

Sendai virus (SeV) is another non-integrative viral vector with a negative single-stranded RNA which is effective for the delivery of genes to different somatic cells. However, due to their continuous replication, removal of SeV vectors from cells is difficult. Moreover, the RNA copy of the viral vector is highly susceptible to the addition of the transgenic protein (Omole and Fakoya, 2018).

The use of viruses, even with non-integrative methods, needs cleansing procedures to remove reprogrammed cells with active replicating viruses. In addition, an innate and adaptive immune response to viral antigens can occur following transplantation of virally reprogrammed cells into patients, because the transplanted cells may be targeted by molecular and cellular cytotoxic pathways (Gois Beghini et al., 2020).

#### 4.2.1 Adenoviruses

Adenoviruses are non-enveloped viruses with double-stranded genomic DNA that prompt temporal expression of the transgene (Rapti et al., 2015). They are non-integrative viruses that serve as valuable expression vectors for the production of iPSCs. The reprogramming efficiency with adenoviral vectors is several times lower than that of lentiviruses or retroviruses and is 0.001%–0.0001% in mice and 0.0002% in human cells (Gois Beghini et al., 2020). Both dividing and non-dividing cells can be infected by adenoviral vectors. The delivery capacity of adenoviruses is limited, and large gene inserts can be transduced using gutless adenoviruses (GLAd), but they are troublesome and require a concomitant virus for co-infection, which makes subsequent cleansing procedures difficult. In this approach, the higher gene delivery efficiency by adenoviruses depends on the coxsackie and adenovirus receptors on the target cells (Fontes and Lakshmipathy, 2013).

#### 4.2.2 Sendai viruses (SeV*)*

Sendai virus is an enveloped, non-pathogenic, single-stranded, and negative-sense RNA virus that belongs to the family Paramyxoviridae. Because SeV does not have a DNA intermediate during its life cycle, it does not incorporate into the host genome; therefore, it can be suitable for generating transgene-free iPSCs. Sendai virus vectors (SeVVs) replicate in the form of a ribonucleoprotein (RNP) complex, and transcription occurs in the cytoplasm of host cells without passing through a DNA phase. The RNP-based replication of SeVs in host cells is reported to enable complete iPSC reprogramming due to the stable expression of reprogramming factors (Hu, 2014; Borgohain et al., 2019).

The first report on the successful reprogramming of human fibroblasts through the SeV-based expression of reprogramming factors was published by the Hasegawa Group in 2009. Instead of wrapping the four common Yamanaka factors, that is, Oct4, Sox2, Klf4, and c-Myc, into an individual virus, the team produced distinct SeV structures for each reprogramming factor. This method was adopted for reasons such as decreased risk of tumorigenesis, enhanced control over stoichiometry of the reprogramming factor, and less harmful effects that are usually associated with simultaneous expression of all four reprogramming factors in a single virus (Schlaeger, 2017). SeV-based reprogramming is relatively efficient and reliable with low work burden along with lack of viral sequences in most high-passage iPSC lines generated using this method. Disadvantages of SeV-based reprogramming include comparatively slow purification of SeV RNA and unavailability of clinical-grade SeV products (Schlaeger et al., 2015).

### 4.3 Non-integrative, non-viral gene delivery

Non-integrating, non-viral systems involve the temporary expression of reprogramming agents using episomal vectors or plasmids which harbor the complementary DNA (cDNA) of reprogramming factors (Oct3/4, Sox2, Klf4, and c-Myc) (Gois Beghini et al., 2020). Since iPSCs generated in this way lack proof of plasmid integration into their genomes, episomal vectors may be the best reprogramming strategy currently available for the clinical use of iPSCs.

#### 4.3.1 Episomal vectors

Episomes are extra-chromosomal DNAs that are able to replicate inside the cell independently of the chromosomal DNA. With the application of episomal vectors as plasmids, the reprogramming factors can be transiently delivered into the somatic cells. In contrast to retroviruses and lentiviruses, episomal vectors are easier to use and provide a reliable gene expression without genomic insertion. Episomal vectors are transiently expressed and therefore may need several transfections which in turn lead to a lower efficiency of reprogramming with this method (Omole and Fakoya, 2018). Episomal vectors are composed of Epstein–Barr virus-derived oriP/EBNA1 viral components. These plasmids facilitate the replication of episomal plasmid DNA in the cells and allow the expression of reprogramming factors for a term long enough for beginning the reprogramming process (Kumar et al., 2018). Since episomal vectors are non-integrative, they are destroyed and/or diluted by cells during multiple cell divisions and repeated passaging over time, thus reducing the risk of insertional mutagenesis as well as the risk of persistent expression of pluripotency factors. However, because these vectors are DNA-based, it is not possible to completely omit the risk of genomic integration (Sridhar et al., 2016). Therefore, it is important to ensure that the method used to create hiPSCs is both efficient enough and safe.

The first episomal reprogramming was reported by the Thomson Group in 2009 (Yu et al., 2009). Various groups have since modified the method, most of which combine common Yamanaka factors with additional reprogramming factors to increase reprogramming efficiency. The additional pathways that are often targeted in this method include the tumor suppressor protein P53 and the genome stability gatekeeper pathway. It has been shown that inhibition of the P53 pathway increases the efficiency of episomal reprogramming (Schlaeger, 2017). Some of the advantages of episomal reprogramming include the diversity of cell types that can be successfully reprogrammed (e.g., skin fibroblasts, blood cells, mesenchymal stem cells, and urinary cells), the simplicity and approximately low cost of the reagents, and the availability of clinical-grade episomal reprogramming protocols (Schlaeger, 2017).

hiPSCs can be reliably generated from fibroblasts, CD34^+^ blood samples, and peripheral blood mononuclear cells with episomal reprogramming. This approach also has the advantage of rapid elimination of reprogramming factors compared to SeV. Because a TP53 hairpin RNA cassette is used in episomal reprogramming, there are concerns about genomic integration in the resulting hiPSC lines. In fact, the rate of aneuploidy with this method is higher than lentiviruses-, SeV-, and RNA-induced hiPSCs but less than that in retrovirally induced hiPSCs (Schlaeger et al., 2015).

#### 4.3.2 The delivery of RNA molecules coding for reprogramming factors

iPSCs have been successfully generated by the direct delivery of synthetic mRNA encoding the reprogramming factors to somatic cells. mRNA technology provides a safer reprogramming procedure than other non-integrative delivery methods, especially because the half-life of RNA molecules is short. However, this same feature makes it necessary to perform frequent transfections to promote the reprogramming procedure. Notably, the methods which are based on RNA are known to be highly immunogenic (Omole and Fakoya, 2018).

However, developments in the RNA technology have improved the efficiency of RNA-mediated iPSC generation, and it has an extremely low likelihood for genomic integration. Moreover, the appearance of the iPSC colonies with the mRNA approach is faster, and the observed aneuploidy rate is much lower than that in other methods. However, there may be some minor issues such as high workload and the requirement for an O_2_-controlled tissue culture incubator (Schlaeger et al., 2015).

##### 4.3.2.1 Synthetic mRNA

Similar to the naturally processed mature mRNA molecules, synthetic mRNA is a single-stranded molecule consisting of a 5′ cap and the untranslated regions (UTRs) encompassing the coding region along with a 3′ poly(A) tail. The mRNA molecule is produced through *in vitro* transcription (IVT) of a linear DNA template. Exogenously administered mRNA can enter the cell either directly through the cell membrane, for example, *via* electroporation or through endocytosis when the mRNA is naked or formulated, which is followed by endosomal escape of the mRNA into the cytosol. The mRNA molecules do not translocate to the nucleus and are not thought to be able to integrate into the genome, since they are less likely to be reverse-transcribed into cDNA molecules inside the cells. The translation process takes place in the cytosol, and proteins translated from synthetic mRNAs are indistinguishable from corresponding proteins produced through endogenous mRNA translation (Beck et al., 2021). Synthetic mRNAs can be used as templates for the synthesis of full-length proteins, protein segments, or peptides (Beck et al., 2021), making them flexible tools for ectopic expression.

##### 4.3.2.2 Self-replicating RNA

Despite its ability to induce pluripotency, synthetic mRNA-based reprogramming needs daily mRNA transfections, enhances cellular stress and cytotoxicity, and induces an innate immune response. As a promising solution to this problem, the self-replicating RNA (srRNA) system continuously expresses reprogramming factors during cell divisions, and thus durable protein expression is achieved (Yoshioka et al., 2013; Steinle et al., 2019). The mRNA delivery can be promoted *via* magnetic forces and mRNA–nanoparticle complexes. Notably, a close interaction occurs between the mRNA–nanoparticle complexes with the target cells because of the transfer of the charged particles, which increases the cellular uptake through endocytosis (Moo-Young, 2019).

**FIGURE 1:**
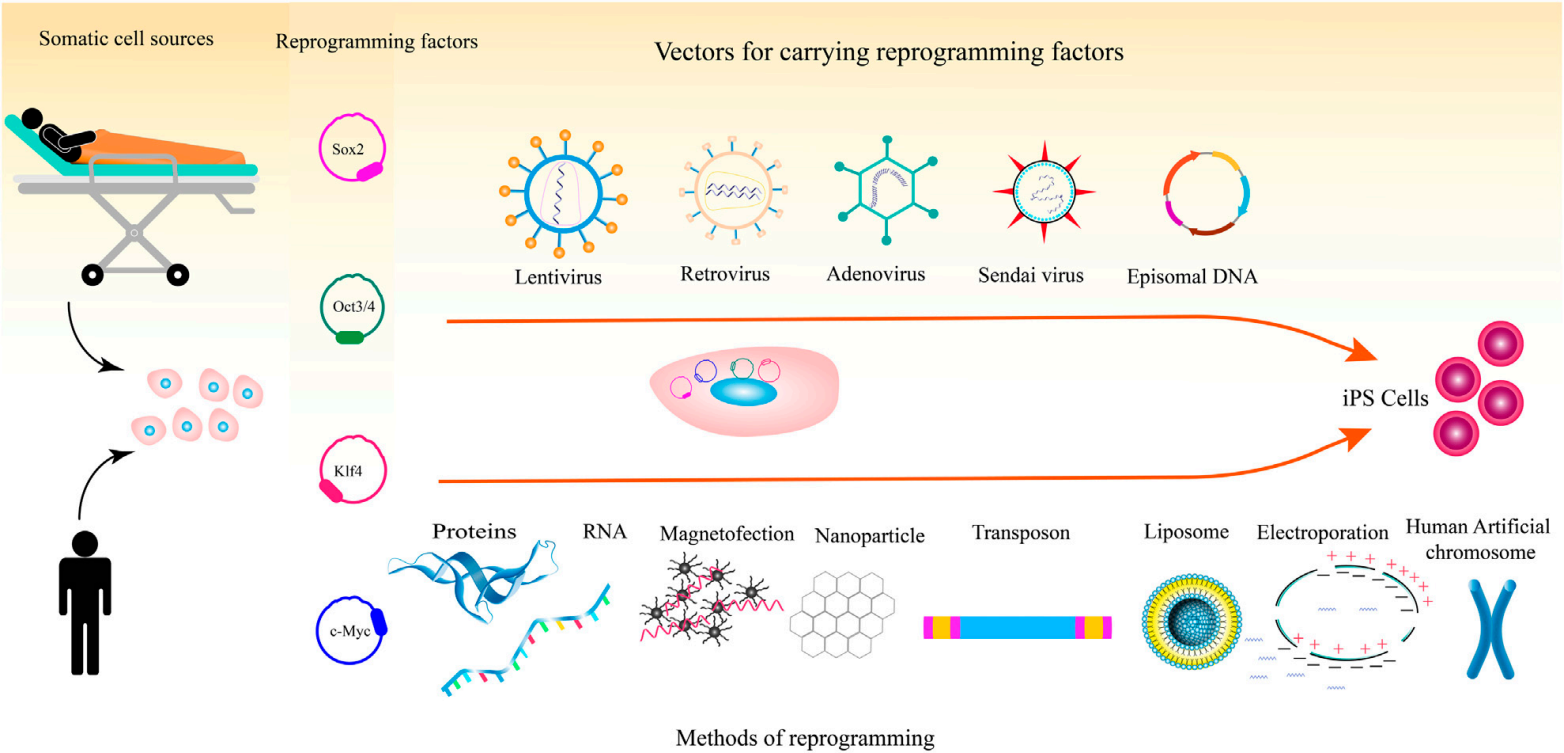
Process of reprogramming somatic cells to iPSCs including somatic cell types, reprogramming factors, and methods for the delivery of the reprogramming factors.

Magnetic nanoparticles have been used to deliver mRNA molecules into cell lines and primary cells. Magnetic nanoparticle-mediated mRNA transfer appears to be an applicable method for reasons such as being inexpensive, controllability, low toxicity to cells, and non-immunogenicity. In 2018, Yamoah et al. showed that hiPSCs and hiPSC-derived cardiomyocytes, that are difficult to transfect, could be efficiently transfected with mRNA molecules using magnetic nanoparticles (Yamoah et al., 2018). Figure 1 illustrates the different methods for the delivery of pluripotency factors to somatic cells.

## 5 The promise of mRNA technology for reprogramming

mRNA technology has various biomedical applications including restoration of the expression of mutated or lost genes, mRNA-based microbial vaccines encoding specific microbial (e.g. bacterial or viral) antigens for inducing immune response, mRNA-based cancer vaccines (both prophylactic and therapeutic), mRNA-based therapeutics for treating various genetic and non-genetic diseases, and mRNA-mediated cellular reprogramming for generating iPSCs (Beck et al., 2021; Damase et al., 2021; Hou et al., 2021). The delivery of mRNA molecules encoding reprogramming factors is a convenient and simple method to generate patient-specific clinical-grade iPSCs without a high risk for genomic integration. In 2010, Yakubov et al. transfected human foreskin fibroblasts with mRNAs of reprogramming factors and produced the first version of iPSCs using IVT-unmodified mRNAs (Borgohain et al., 2019). The mRNA reprogramming technology has some advantages over other reprogramming methods due to features such as high mRNA production speed, high efficiency with which iPSCs are generated in this way, and ability to control the dose and stoichiometry of the reprogramming factor (Warren and Lin, 2019).

## 6 Cell fate reprogramming with small molecules

The possibility of genetic changes and the risk of tumorigenesis associated with reprogramming using integrative strategies has encouraged scientists to investigate the application of safer reprogramming methods. For this purpose, the use of certain small molecules has been proposed to reprogram somatic cells to PSCs. Several small molecules have been reported to increase the reprogramming efficiency of iPSCs that are produced by other methods. In few cases, it has been suggested that iPSCs could be generated with combinations of small molecules alone. These so-called chemically induced iPSCs were first generated in 2013 by Hou et al. by substituting Oct4 with the small molecule compound forskolin (Hou et al., 2013).

The important superiorities of this strategy compared to other methods include cost effectiveness, higher safety, easy delivery due to a high level of cell permeability, the possibility to control the dose and timing of administration, the possibility of their combination with other reprogramming factors to achieve synergistic effects, the ease of synthesis that allows mass production, and the least residual effects on the genome. In addition, the application of small molecules removes the risk of inappropriate expression of oncogenes associated with integrative approaches because it eliminates the need to use oncogenes.

A wide variety of small molecules used for reprogramming fall into one of the three categories: signaling modifiers, epigenetic modifiers, and metabolic modifiers. The largest category consists of small molecules with signaling activity, including RepSox, forskolin, AM 580, SB431542, tranilast, A 83–01, bromodeoxyuridine (BrdU), PD0325901, thiazovivin, and cyclic pifithrin-α, which contain compounds inhibiting TGF-β and Hedgehog signaling pathways, both of which are involved in cell differentiation. The second category includes the epigenetic modifiers valproic acid, parnate (Tranylcypromine), 3-deazaneplanocin A, EPZ004777, NaB, trichostatin A, 5-AZA-dC, and SGC0946 (Masuda et al., 2013; Biswas and Jiang, 2016). Most of these epigenetic modifiers are inhibitors of methyltransferases (histone methyltransferases, DNA methyltransferases, and histone deacetylases), which regulate the chromatin structure. Others exhibit a dual activity (i.e., inducers and/or inhibitors of histone deacetylases) or simultaneously inhibit two chromatin methyltransferase enzymes, which results in a decrease in the level of DNA methylation and increase in the DNA available for transcription. Finally, the metabolic modifier category includes CHIR99021, lithium carbonate, and lithium chloride, which changes the metabolism from oxidative phosphorylation to glycolysis, mainly by inhibiting the GSK3 enzyme (Kim et al., 2020; Knyazer et al., 2021; Lange et al., 2022).

## 7 Function of microRNAs in reprogramming of somatic cells to iPSCs

Small non-coding RNAs known as microRNAs (miRNAs) promote the degradation or translational repression of target mRNAs by binding to partially complementary regions on their target transcripts, thereby controlling the majority of biological and developmental pathways (Bhaskaran and Mohan, 2014; Mohr and Mott, 2015; Bereimipour et al., 2021; Mohammadi et al., 2021). The role of miRNAs in the reprogramming process has been demonstrated in numerous studies (Moradi et al., 2014; Li et al., 2017; Nishimura et al., 2017; Mong et al., 2020). For example, miR-302/367 and miR-372 have been shown to increase the efficiency of hiPSC production by inhibiting transforming growth factor-β (TGF-β)-induced epithelial–mesenchymal transition (EMT) and promoting mesenchymal–epithelial transition (MET) during reprogramming (Mochiduki and Okita, 2012). The forced expression of ESC-specific miRNAs from the miR-290 cluster was found to substitute for c-Myc during iPSC reprogramming (Yang et al., 2011). Since decreased p53 expression can considerably enhance reprogramming efficiency, researchers used miR-29a removal, which extremely reduced p53 levels and significantly increased reprogramming efficiency. Inhibition of c-Myc-targeting miRNAs, that is, miR-21 and miR-29a, nearly tripled the reprogramming efficiency, indicating that miRNAs enriched in starting somatic cells act as barriers to reprogramming (Yang et al., 2011). The miR-302/367 cluster, which is highly expressed in ESCs and iPSCs (Lin et al., 2011; Moradi et al., 2017), is directly activated by Oct4 and Sox2, two essential factors for iPSC generation. It has been reported that human and mouse somatic cells can be rapidly reprogrammed to iPSCs solely by overexpression of the miR-302/367 cluster in the absence of all OSKM reprogramming factors (Lin et al., 2011), although other groups could not reproduce this finding (reviewed in Moradi et al. (2014)). However, it is certain that the miR-302/367 cluster remarkably enhances the efficiency of iPSC generation (reviewed in Moradi et al. (2014)). The main reason why miRNA-mediated reprogramming is rapid is probably related to the nature of miRNAs, because miRNA expression does not involve protein expression (i.e., miRNAs are non-coding RNAs) and therefore leads to a rapid decrease in target mRNA translation. Finally, miRNAs typically target hundreds of mRNA transcripts that, in turn, harmonize the expression of a large number of various proteins and can quickly force a major phenotypic alteration in cellular identity (Anokye-Danso et al., 2011).

## 8 Characterization and quality assessment of iPSCs

After generation, iPSCs must first be characterized. This procedure is needed to confirm the pluripotency of iPSCs generated with different methods. The emergence of the first iPSC colonies is usually confirmed under a microscope based on the ESC-like morphology, expandable colonies, and positive staining for alkaline phosphatase staining. Once fully reprogrammed iPSC colonies are formed, they can be physically picked up and transferred to new culture dishes for further characterization. These characterizations include the analysis of the expression of various pluripotency markers at the mRNA (qRT-PCR and microarray or RNA-sequencing) and protein levels (flow cytometry and immunostaining), assessment of multi-lineage differentiation potential using spontaneous (embryoid body formation) and directed differentiation toward various mesodermal, ectodermal, and endodermal lineages, and the ability to form teratomas, benign tumors that are formed when truly PSCs are injected into immunocompromised mice (Bharathan et al., 2017; Guo et al., 2020; Shen et al., 2022).

Depending on the reprogramming method and the efficiency of iPSC production, ESC-like colonies with variable quality are expected to form. Some iPSC colonies are composed of fully reprogrammed, high-quality iPSCs whereas others are only partially reprogrammed. The partially reprogrammed iPSCs usually display defects in the establishment of a self-sustained gene regulatory circuitry typical of PSCs. Such colonies may also show some deficiencies at the morphology, expandability, and/or differentiation propensity levels, which make them less useful for PSC biomedical applications. Since somatic cell reprogramming forms a large number of colonies, one can always select and pick up iPSC colonies that appear fully reprogrammed judging from the ESC-like morphology and doubling time kinetics, etc., removing the need to spend time on partially reprogrammed iPSC colonies. Finally, it would be worthwhile to analyze the genomic integrity of the obtained iPSCs using karyotype analysis and whole-genome sequencing (Lund et al., 2012; Poetsch et al., 2022). This analysis is of critical importance when iPSCs are intended to be used for clinical purposes.

## 9 Induction of pluripotency and its molecular mechanisms

During cellular reprogramming, the expression of particular genes of the primary cell types changes *via* various epigenetic alterations, without altering the genomic sequences of the genes. Since endogenous gene expression and epigenetic modifications govern cell fate decisions, the exogenous expression of transcription factors induces substantial changes in the regulation of the somatic cell fate and gradually converts it into an ESC-like cell state (Han et al., 2021b). The reprogramming process involves random and hierarchical steps, which begins with increasing the expression of genes that control DNA replication and cell division and suppressing the expression of genes responsible for cell adhesion and cell-to-cell contact. In the first phase of reprogramming, the combination of c-Myc with histone acetyltransferase complexes leads to a global induction of histone acetylation, enabling the binding of exogenous Oct4 and Sox2 to target sequences on DNA. Klf4 plays a dual role in the early and random stages of the reprogramming process, promoting the suppression of a large number of genes specific to the intermediate reprogramming cells and inducing the activation of the genes associated with pluripotency. Sox2 appears to be involved in all stages of the reprogramming process, particularly in the hierarchical stage. Finally, Oct4, as the key, indispensable component of the reprogramming process, reorganizes chromatin, thereby activating pluripotency gene expression (Kulcenty et al., 2015).

## 10 Reprogramming and the associated epigenetic changes

### 10.1 DNA methylation during reprogramming

DNA methylation is one of the crucially important epigenetic mechanisms that has pivotal roles in diverse processes including cell division, normal development, proper chromosome regulation, and cell differentiation, and is known to serve as an important epigenetic barrier during the reprogramming process (Smith and Meissner, 2013; Gomes et al., 2017). Since demethylation of cytosines at the promoter regions of pluripotency-associated genes has been observed to be indispensable for the expression of genes necessary for reprogramming, strategies such as inducing certain DNA demethylase enzymes or administration of specific siRNAs against DNA methyltransferases have been used to inhibit global DNA methylation and improve the reprogramming process (Gomes et al., 2017). Interestingly, while induction of global demethylation helps increase the reprogramming efficiency of somatic cells to pluripotency, human iPSCs themselves, similar to established human ESCs, have a hypermethylated genome. In fact, cumulative evidence has revealed that while a global hypomethylation is required for the earlier stages of somatic cell reprogramming to erase the somatic cell memory, genome-wide DNA methylation is substantially enhanced at the late phase of iPSC establishment by maintenance DNA methyltransferases (Doi et al., 2009; Bhutani et al., 2010). Importantly, it has been found that incomplete DNA methylation of iPSCs leads to the retention of a somatic cell memory in the reprogrammed iPSCs, which may impair the differentiation capacity of the cells (Ohi et al., 2011).

### 10.2 Histone changes during reprogramming

In starting cells infected with OSKM factors that have gone through multiple cell divisions, active H3K4 dimethylation respreads at more than a thousand target loci containing promoters and enhancers of many pluripotency genes prior to transcriptional activation, while the H3K4 trimethylation (H3K4me3) marks occur only locally. During the reprogramming process, H3K4me3 levels show two distinct phases of increase. The first increase in H3K4me3 marks occurs due to the genes associated with the ESC state, whereas the second increase takes place during the reprogramming to pluripotency (Qin et al., 2016). As an epigenetic mark that is mainly associated with gene repression, H3K9 methylation acts as a major obstacle to reprogramming. Induction of certain histone demethylases or using siRNAs against H3K9 methyltransferases has been found to enhance reprogramming efficiency (Wang et al., 2011; Vidal et al., 2020). Onder et al. showed that H3K79 dimethylation (H3K79me2) prevented repression of lineage-specific programs and acted as a barrier during reprogramming. Removal of H3K36me2/3 marks, which are a barrier during reprogramming, increases the efficiency of reprogramming (Ebrahimi, 2015). The histone modifying enzyme Jhdm1b (Kdm2b) has been observed to promote iPSC formation by H3K36 demethylation. Finally, histone deacetylation by histone deacetylases (HDACs) facilitates the formation of heterochromatin, making chromatin unavailable for transcription. Inhibition of HDAC enzymes using small molecules such as valproic acid, trichostatin A, supervilanilide hydroxamic acid, and sodium butyrate has been reported to significantly increase the conversion of somatic cells into iPSCs (Haridhasapavalan et al., 2020). Taken together, these collective findings highlight the critically important roles that are played by various epigenetic modifiers, DNA methylation, and diverse histone marks over the course of reprogramming to pluripotency.

## 11 Application of iPSCs in COVID-19 research

COVID-19, which has been started since December 2019 by SARS-CoV-2, has now become a global pandemic (Yu et al., 2020). Since 1) our knowledge of the pathogenesis, transmission, mechanisms underlying infection, and host responses to the SARS-CoV-2 is limited, 2) there is still no specific drug to treat SARS-CoV-2, and 3) simulation of host–virus interaction *in vitro* is different from that in the human body, modeling SARS-CoV-2 infection in human tissue models is essential for establishing a human laboratory COVID-19 model to understand the SARS-CoV-2 infection process and drug screening (Chen et al., 2020; Rando, 2021; Chakrabarty et al., 2022).

Several modeling platforms have been utilized for the investigation of SARS-CoV-2 infection. Human biopsies, animal models, and iPSCs have been the major platforms for modeling COVID-19. Human biopsies provide valuable information regarding the pathology of COVID-19. For example, several studies have been conducted through needle biopsies of the lung, liver, and heart cores in patients who died due to COVID-19. These studies have revealed how SARS-CoV-2 interacts with its target tissues and how it creates injuries in the parenchyma of the tissues (Tian et al., 2020; Giani and Chen, 2021). The limitations of using biopsies is the limited number of available samples, the short storage time of biopsies outside the body, and the need to get permission from the deceased’s family immediately after death (Tian et al., 2020; Giani and Chen, 2021).

In addition to human biopsies, several animal models have been used for SARS-CoV-2 modeling, among which mice are the most widely used species. The problem with using mice to model SARS-CoV-2 is that they show resistance to SARS-CoV-2, which can be overcome by introducing the human ACE2 receptor (hACE2) into the cells *via* an adenovirus. Ferrets, dogs, and cats that are susceptible to SARS-CoV-2 have also been used to model SARS-CoV-2 (Larijani et al., 2021). Modeling of SARS-CoV-2 infection has also been performed with golden Syrian hamsters which developed clinical symptoms of the disease, transmitted the virus through aerosols to other animals, and induced neutralizing antibody responses, which led to the identification of therapeutic strategies against SARS-CoV-2 infection. The disadvantages of animal models for COVID-19 are the difficulty of scaling, the lack of genetic tractability, and limited access to the animals (Leist, 2022).

**FIGURE 2:**
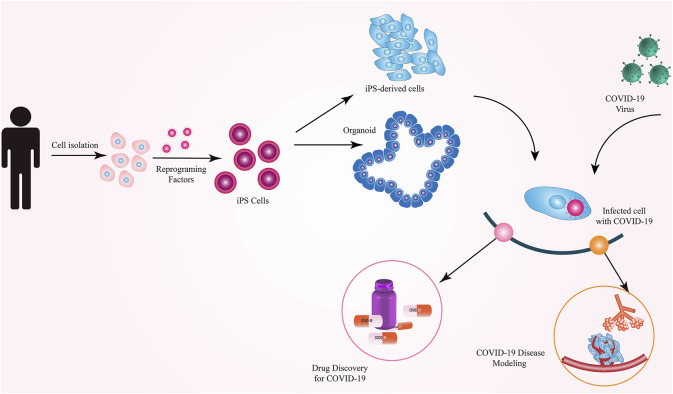
Applications of iPSCs in COVID-19 research.

iPSCs have been reported to be the most widely used cell type used to model COVID-19 (Cuevas-Gonzalez et al., 2021). Although both iPSCs and ESCs are expected to be similarly applicable for COVID-19 modeling, iPSCs have gained more attention for this purpose. Notably, iPSCs generated from COVID-19 patients or specific populations may provide a more realistic environment for such modeling studies because it is known that the genetic background and ethnicity play a role in the susceptibility to, and severity of, COVID-19 (Elhabyan et al., 2020; Pan et al., 2020). Such patient- or population-specific iPSCs would serve as a more reliable platform for COVID-19 modeling and can offer the possibility to present person- or population-specific treatments. The limitations of using iPSCs in SARS-CoV-2 modeling include the possibility of retaining the somatic cell memory in iPSCs and, therefore, the possibility of biased differentiation toward the fate of the starting cell type (instead of efficient differentiation toward the lung epithelial cells for COVID-19 modeling). Furthermore, the presence of embryo-like characteristics in differentiated cells derived from iPSCs, the time-consuming nature, and high costs of reprogramming are other challenges of modeling COVID-19 using iPSCs. Different types of cells and organoids derived from hiPSCs are excellent platforms for studying viral infection processes, pathogenesis, virus–host interrelationships, and drug testing (Luo et al., 2021) (Figure 2).

## 12 Experimental models of SARS-CoV-2

### 12.1 Cellular models

In 2020, Surendran et al. used hiPSC-derived lung epithelial cells as a human system to study SARS-CoV-2 infection. This approach could be a new system for modeling the disease and screening the effectiveness of drugs for treating COVID-19 (Surendran et al., 2020). Using iPSC-derived alveolar type 2 epithelial cells (AT2s) and modeling SARS-CoV-2 infection of the alveolar epithelium, Huang et al. developed a human model for SARS-CoV-2 infection and evaluation of drug effectiveness (Huang et al., 2020). The leading cause of death from COVID-19 is extensive alveolar damage and pneumonia. The SARS-CoV- 2 not only uses the receptor ACE2 (angiotensin-converting enzyme receptor type 2) to enter the target cells but also uses various mechanisms such as the induction of types I and III interferon (IFN-III) production to enter the host cells, which increases the transmissibility of SARS-CoV-2. With the aim of recognizing the innate immune mechanisms modulated in the target cell in response to virus entry, Katsura et al., infected 3D alveolosphere cultures of primary human AT2s, that is, the stem cells of the distal alveolar zone, with SARS-CoV-2 and monitored cellular and molecular responses over time. In this study, they showed that the AT2 alveolosphere system based on human stem cells is a unique model for the understanding of COVID-19 and other respiratory diseases (Katsura et al., 2020). In a study conducted by Marchiano et al., hPSC-derived cardiomyocytes and hPSC-derived smooth muscle cells were used to investigate the causes of heart complications in COVID-19 patients (Marchiano et al., 2021). In addition, Wong et al. showed the ability of hPSC-derived cardiomyocytes to be used as a model for assessing the susceptibility of cardiomyocytes to SARS-CoV-2 infection and the study of the fundamental mechanisms of myocardial damage, including direct cytopathogenic effects of the virus and inflammatory responses of cytokine/chemokine to SARS-CoV-2. They also revealed that the hPSC-derived cardiomyocytes could be used as a reliable model to study antiviral drugs and evaluate their efficacy (Wong et al., 2020). Monteil et al. used human capillary organoids derived from iPSCs to test whether human recombinant soluble ACE2 (hrsACE2) could inhibit SARS-CoV-2 infection (Monteil et al., 2020). Wang et al. developed a human iPSC-derived airway epithelial platform to study the response of airway cells to SARS‐CoV‐2 infection and to evaluate the effectiveness of the antiviral drug remdesivir on SARS‐CoV‐2 infection. They first showed that the SARS-CoV-2 entry factors ACE2 and TMPRSS2 were expressed in multiple iPSC airway epithelial cell lineages. Reduction in viral replication in treatment with remdesivir indicated that iPSC-derived airway epithelial cells are a valuable model for evaluating the effect of drugs on COVID‐19 (Wang et al., 2022). Abo et al. used human iPSC-derived alveolar and airway epithelial cells as a physiological model to study SARS-CoV-2 infection, which allows for the analysis of several aspects of SARS-CoV-2 infection such as viral entry, cellular response to the virus, and the viral replication (Abo, 2020). Table 1 indicates a summary of cellular models of COVID-19 using pluripotent stem cells.

**TABLE 1:** List of cell models of COVID-19 modeling using pluripotent stem cells.

Cell model	Cell type used	Reference
Pancreatic endocrine cells, cardiomyocytes, microglia, endothelial cells, macrophages, dopaminergic neurons, and cortical neurons derived from human pluripotent stem cells	Human pluripotent stem cells	(Yang et al., 2020)
iPSC-derived airway epithelial platform	Human-induced pluripotent stem cells	(Wang et al., 2022)
Human iPSC-derived alveolar and airway epithelial cells	Human-induced pluripotent stem cells	(Abo, 2020)
Human iPSC-derived lung epithelial cells	Human-induced pluripotent stem cells	(Surendran et al., 2020)
Pluripotent stem cell-derived human lung alveolar type 2 cells	Human-induced pluripotent stem cells	(Huang et al., 2020)
Human pluripotent stem cell-derived neural cells	Human pluripotent stem cells	(Jacob et al., 2020)
Human pluripotent stem cell-derived cardiomyocytes	Human pluripotent stem cells	(Marchiano et al., 2021)
Human iPSC-derived cardiomyocytes	Human pluripotent stem cells	(Wong et al., 2020)

Two-dimensional cell cultures of various cell line models such as Caco-2, Calu-3, HEK293T, Vero, and Huh7 appear to more accurately simulate the pathophysiology of SARS-CoV-2 infection in the apical membrane of cells (where SARS-CoV-2 infection occurs), compared to 3D cultures such as organoids (Takayama, 2020; Larijani et al., 2021). Higher virus titers have been observed in 2D constructs compared to 3D organoids, hence, they appear more useful for testing antiviral agents. Moreover, because the apical portion of the cells is exposed to air in 2D cultures (in contrast to organoids where the cellular apex is inside), 2D cultures are more suitable for studying virus pathogenesis (Larijani et al., 2021).

### 12.2 Organoid models

Despite the usefulness of the cellular models in studies of SARS-CoV-2 infection, they do not exactly mimic human physiological conditions (Takayama, 2020). On the contrary, organoids have a higher advantage in disease modeling compared to *in vitro* as well as *in vivo* models, which can be attributed to the higher speed of this modeling strategy. Organoids enable the comparison of different SAR-CoV-2 strains and their susceptibility to vaccines and drugs (Larijani et al., 2021). Finally, another advantage of organoids is their ability to provide reliable molecular assessment of differences in viral susceptibility in different individuals and races (Larijani et al., 2021).

Because the lungs are the organs most affected by COVID-19, establishing an *in vitro* lung model with similar function to the lungs of living organisms is critical to evaluating the factors influencing COVID-19 treatment. Suzuki et al. were able to use human bronchial epithelial cells to create human bronchial organoids for use in COVID-19 research. According to research conducted by Suzuki and his group, these cells were infected by COVID-19 and the virus replicated in these cells. They also evaluated the effectiveness of TMPRSS2 inhibitor camostat on the infection of SARS-CoV-2 in human bronchial organoids and showed its beneficial effect on these cells (Suzuki, 2020). Using hPSCs, Han et al. developed an organoid model of the lung to evaluate the efficacy of drugs against SARS-CoV-2 infection. Using hPSC-derived lung organoids, they showed that candidate drugs for the treatment of COVID-19, including imatinib and mycophenolic acid, prevented the cellular entry of SARS-CoV-2, which manifested itself by reducing SARS-CoV-2 infection of hPSC-derived lung organoids (Han, 2020). In a separate study, Han et al. generated colonic organoids using hPSCs to analyze the gastrointestinal manifestations observed in patients with COVID-19. They evaluated the response of colon cells to SARS-CoV-2 infection and the effectiveness of a number of drugs that inhibit SARS-CoV-2 entry. They found that colon cells, especially enterocytes, were susceptible to SARS-CoV-2 infection due to ACE2 expression. They also showed that imatinib, mycophenolic acid, and quinacrine dihydrochloride inhibited SARS-CoV-2 infection in hPSC-derived colonic organoids (Han et al., 2021c). Yang et al. presented a comprehensive platform consisting of eight cell types, including pancreatic endocrine cells, cardiomyocytes, microglia, endothelial cells, macrophages, dopaminergic and cortical neurons, and liver organoids derived from hPSCs to model COVID-19, thereby understanding the pathology of the disease and the cellular responses of various human tissues to SARS-CoV-2 infection. Using this platform, they revealed that liver organoids, pancreatic α and β cells, cardiomyocytes, and dopaminergic neurons are susceptible to SARS-CoV-2 infection (Yang et al., 2020). Abo et al. used iPSC-derived human intestinal organoids to show that iPSC-derived intestinal organoids could be used to model SARS-CoV- infection, which is due to their transcriptional similarity to the primary gastrointestinal epithelium and the expression of ACE2 and TMPRSS2 (Abo, 2020). Duan et al. used *in vitro* and *in vivo* (i.e., humanized mice carrying hPSC-COs) models of hPSC-derived colonic organoids to evaluate the allowance of different types of colonic cells for SARS-CoV-2 entry and to screen FDA-approved drugs against viral infection. Using this platform, they found that the expression of the ACE2 viral input receptor occurs in a variety of hESC-derived colon cells. They also showed that SARS-CoV-2 infection was blocked *in vitro* and *in vivo* by mycophenolic acid and quinacrine dihydrochloride (Duan et al., 2020). Krüger et al. used hPSC-derived intestinal organoids to understand the pathogenesis of SARS-CoV-2 and to evaluate the efficacy of a particular drug on the gastrointestinal tract of COVID-19 patients. They found that most cell types in hPSC-derived intestinal organoids, with the exception of goblet cells, became infected with SARS-CoV-2. They concluded that different cell types in gastrointestinal tissues are differentially susceptible to this infection due to the expression of SARS-CoV-2 entry factors, namely, ACE2 and TMPRSS2. They also demonstrated the effectiveness of remdesivir in controlling SARS-CoV-2 infection (Krüger et al., 2021). Mithal et al. developed two organoid models, proximal intestinal and hiPSC-derived colonic organoids to model SARS-CoV-2 infection in different intestinal epithelia and study host responses to viral infection. The results of their research showed that due to the infection of both proximal and distal human intestinal organoids with SARS-CoV-2, gastrointestinal cells were suitable hosts for SARS-CoV-2 (Mithal et al., 2021). In a study by Jacob et al., a bed of monolayer neurons, microglia, astrocytes, and specific regional brain organoids derived from hiPSCs was applied to assess their susceptibility to SARS-CoV-2 infection and evaluate the tropism of SARS-CoV-2 to different brain cells (Jacob et al., 2020). Ramani et al. used brain organoids and neurons derived from iPSCs to show the detrimental effects of SARS‐CoV‐2 infection on the central nervous system, demonstrating the ability of iPSC-derived organoids to model central nervous system pathology induced by COVID‐19 (Ramani et al., 2020). Despite their advantages, organoids suffer from certain limitations. The lack of immune cells is the main limitation of organoids in the study of SARS-CoV-2 infection, compared to animal models (Larijani et al., 2021). Lack of blood vessels and inter-organ communication are other limitations of organoids (Han et al., 2022). Another weakness of organoids is their inability to reproduce systemic symptoms related to whole-body responses to viral infection (Takayama, 2020). Table 2 summarizes organoid models that have so far been used for COVID-19 modeling using pluripotent stem cells.

**TABLE 2:** List of organoid models for COVID-19 modeling using pluripotent stem cells.

Organoid type	Cell type used	Reference
Human bronchial organoids	Human bronchial epithelial cells	(Suzuki, 2020)
Human lung organoids	Human pluripotent stem cells	(Han, 2020)
Human colonic organoids	Human pluripotent stem cells	(Han et al., 2021c)
Human liver organoids	Human pluripotent stem cells	(Yang et al., 2020)
Human intestinal organoids	Human-induced pluripotent stem cells	(Abo, 2020)
Human colonic organoids	Human pluripotent stem cells	(Duan et al., 2020)
Human intestinal organoids	Human pluripotent stem cells	(Krüger et al., 2021)
Proximal intestinal and colonic organoids	Human-induced pluripotent stem cells	(Mithal et al., 2021)
Human brain organoids	Human pluripotent stem cells	(Jacob et al., 2020)
Human brain organoids	Human-induced pluripotent stem cells	(Ramani et al., 2020)
Human capillary organoids	Human-induced pluripotent stem cells	(Monteil et al., 2020)

## 13 Conclusion

By providing an unlimited source of PSCs that can be differentiated into different cell types, iPSCs have emerged as a problem-solving key for 1) treating diseases that require a specific cell type to replace a lost or damaged tissue, 2) advancing many clinical studies that are otherwise difficult or impossible to conduct, 3) creating cell and tissue banks for patients in times of emergency, and 4) measuring the effectiveness of new or modified drugs. Despite the extraordinary capabilities of iPSCs, their production and use are still challenging. The production of (clinical-grade) iPSCs is not easy, which explains why numerous methodologies have so far been used to generate them. In fact, researchers are still discovering and evaluating new methods of iPSC generation. Concerns associated with different reprogramming methods include whether the reprogramming agents applied are mutagenic or not, cost effectiveness for mass production, and the efficiency of reprogramming. The use of integrative approaches such as lentiviral and retroviral vectors, despite their high efficiency in reprogramming somatic cells, carries the risk of genetic modification. The use of non-integrative viral methods such as adenoviruses and Sendai viruses are also associated with the difficult removal of viral agents. In fact, the use of viral vectors generally requires the removal of active viruses. The use of episomal vectors also has disadvantages such as low efficiency and high risk of aneuploidy. In general, DNA-based methods carry the risk of genetic modification and may not be suitable for clinical applications. The use of RNA to reprogram somatic cells into iPSCs has shown a higher potential among non-integrative methods due to its advantages such as cytosolic expression, high efficiency, and low risk of chromosomal alterations. The use of nanoparticles in various shapes and combinations in inducing pluripotency helps to make the RNA-based reprogramming process more efficient. The use of self-replicating RNA, as a type of mRNA that provides an adequate and sustainable expression of pluripotent factors, has been at the forefront of the latest research methods for the production of iPSCs. COVID-19 is the newest and most serious pandemic that has taken countless lives. There is no definitive treatment for COVID-19 and a large number of studies are underway to discover the hidden dimensions of this disease and to understand its pathogenic mechanisms in order to enable the discovery of new effective drugs. iPSCs have become a useful and valuable tool for COVID-19 modeling and evaluating the effectiveness and possible side effects of possible drugs effective in the treatment of COVID-19. iPSCs have provided the possibility of conducting various COVID-19 research studies *in vitro*, and so far, many studies have used iPSCs for modeling and interrogating the mechanisms of SARS-CoV-2 infection. Despite extensive research efforts in the field of iPSCs, the generation of safe, highly efficient, cost-effective, and publicly available iPSCs still needs further research.
